# Apolipoprotein E polymorphisms contribute to statin response in Chinese ASCVD patients with dyslipidemia

**DOI:** 10.1186/s12944-019-1069-5

**Published:** 2019-06-01

**Authors:** Lei Zhang, Siying He, Zuhua Li, Xuedong Gan, Siwei Li, Xiaohuan Cheng, Na Yang, Fang Zheng

**Affiliations:** 1 Center for Gene Diagnosis, Zhongnan Hospital of Wuhan University, Donghu Road 169#, Wuhan, 430071 China; 2 Department of Cardiology, Zhongnan Hospital of Wuhan University, Donghu Road 169#, Wuhan, 430071 China

**Keywords:** Apolipoprotein E, Polymorphism, Statins response, KASP

## Abstract

**Background:**

Apolipoprotein E (ApoE) plays an important role in lipid metabolism and clearance. Statins are the most common drugs used to modulate the lipid profile in the clinic therapy; the associations between ApoE polymorphisms and statin response to lipids were inconsistent in previous studies among different ethnicities. Our study aimed to demonstrate the relationships among the statins response and the ApoE gene common polymorphisms and lifestyle risk factors in Chinese arteriosclerotic cardiovascular disease (ASCVD) patients with dyslipidemia.

**Methods:**

A total of 1002 dyslipidemia ASCVD patients were recruited in this study, including 311 patients with a history of type 2 diabetes mellitus (T2DM). These patients were all treated with drugs atorvastatin (10 mg/d) or rosuvastatin (5 mg/d) for at least 4 weeks and genotyped for ApoE e2/e3/e4 alleles, using Kompetitive Allele Specific PCR (KASP) and Sanger sequencing. The plasma lipids levels were determined before and after statins treatment.

**Results:**

The results of ApoE genotyping with KASP method were consistent with the sequencing analysis. In the total 1002 patients, the E2 phenotypes (e2/e3, e2/e2) had significant lower low-density lipoprotein cholesterol (LDL-C) baseline levels than subjects with E3 (e3/e3, e2/e4) and E4 (e3/e4, e4/e4) phenotypes (*P* = 0.007, 0.005, respectively), and E2 phenotypes had the highest triglyceride (TG) baseline levels. To statins treatment, E2 phenotypes had a better response in TG, Total cholesterol (TC) and LDL-C reduction percentage compared with other phenotypes, and smoking/alcohol drinking status also had a significant influence on statins response of LDL-C lowering. No significant difference was found in the effects of lipids decreasing between atorvastatin and rosuvastatin drugs in all patients.

**Conclusions:**

We developed the KASP technique for the ApoE genotyping, and demonstrated ApoE polymorphisms interacted with smoking/drinking to influence the declining extent of TG, TC and LDL-C levels after statins therapy in Chinese dyslipidemia ASCVD patients. These discoveries developed our cognition with the genetic polymorphisms effects on statin response, which should be taken more seriously in smoking/drinking E4 amino acid isoform carriers.

## Background

Statins are widely used to reduce the LDL-C of the circulation and improve the lipid profile in hypercholesterolemia and other cardiovascular diseases [1]. However, the effectiveness of statin therapy may be influenced by several factors: treatment adherence, age, gender and even genetic variability of each patient [2, 3]. Because of this, pharmacogenetic studies of lipid-lowering response to statins were established in investigating the associations between genetic variations and therapeutic response of statins and involved other related processes like metabolism and pharmacokinetics [4, 5]. Since the association could be partially influenced by the ethnicity of individuals, numbers of patients and the underlying diseases, the previous research didn’t always get results in accordance and haven’t included lifestyle risk factors as potential confounders.

The Apolipoprotein E (ApoE) gene in human located on the chromosome 19q13.2, the differences of amino acid at the location of 112 and 158 caused the three major alleles: e2 (Cys112 and Cys158), e3 (Cys112 and Arg158), e4 (Arg112 and Arg158), because of the two single nucleotide polymorphisms (SNPs) (rs429358 T > C, rs7412 C > T). The combination of three alleles formed six ApoE genotypes: 3 heterozygous (e2/e3, e2/e4 and e3/e4), and 3 homozygous (e2/e2, e3/e3, e4/e4) [6]. These ApoE gene polymorphisms of individuals affect the lipoproteins clearing and the lipid profile, which is also an important candidate susceptible gene for type2 Diabetes (T2DM) and/or arteriosclerotic cardiovascular disease (ASCVD) [7–10]. But the results of the association between ApoE polymorphisms and the risk of coronary artery disease (CAD) in different populations were conflicted [11–14]. Furthermore, the E4 phenotype has shown the association with the lower response to the statin therapy in some studies [15–17], while there was research reported ApoE polymorphisms showed no significant effect on the response of statin in Chinese patients with hyperlipidemia [18] .

Thus, it is necessary to conduct a study to investigate the effect of statins to lower the lipids profile in the Chinese population carrying different ApoE genotypes and evaluate the interaction on statins response among ApoE variants, living habits and history of diseases.

In addition, though kinds of technique had been applied for ApoE genotyping in the clinic for individual therapy of statin, such as TaqMan assay [19] and high resolution melting (HRM) technique, etc., an efficient and economic genotyping method is required in clinical application [20]. In this research, an efficient and economic KASP technique was successfully established to validate the ApoE polymorphisms, which based on allele-specific primer extension [21] and fluorescence-based genotyping technology.

## Methods

### Subjects

The 1002 patients involved in this study were collected in Zhongnan Hospital of Wuhan University, Central Hospital of Wuhan and Shandong Provincial Hospital from November 2016 to December 2017. All patients in this study were more than 18 years old and with ASCVD. Patients who had the baseline levels of TC ≥ 3.1 mmol/L or LDL-C ≥ 1.8 mmol/L and have been treated with statins (Atorvastatin 10 mg/d or Rosuvastatin 5 mg/d) for at least 4 weeks [22, 23], were recruited. In addition, the baseline levels of TG and HDL-C of participants were 1.87 (1.25, 2.60) for TG and 1.09 (0.94, 1.30) for HDL-C. The patients with hepatic, kidney and endocrinological or malignant diseases were excluded.

The informed content was obtained and the study got the approval by the Medical Ethics Committee of Zhongnan Hospital of Wuhan University.

### Lipid profiling

Blood samples were obtained by venipuncture following 12 h overnight fast. Two measurements of lipids levels were obtained for each sample: prior to treatment and ≥ 4 weeks post treatment (Atorvastatin 10 mg/d or Rosuvastatin 5 mg/d). The lipids profiles of TG, TC, LDL-C and HDL-C were determined on the automatic clinical chemistry analyzer in the core laboratory. Clinical data of study participants were collected, included age, sex, blood pressure, and histories of smoking, alcohol intake, diabetes mellitus, and lipid-lowering drug treatment (Table 1).

**Table 1:** Clinical characteristics of the study participants

Characteristics	*N* = 1002
Age, years	65 (57–75)
Male/female	620/382
Systolic blood pressure (mmHg)	132 (120–145)
Diastolic blood pressure (mmHg)	78 (70–85)
Smoking/non-smoking	303/699
Drinking/non-drinking	237/765
T2DM/non-T2DM	311/691
Atorvastatin/Rosuvastatin	575/427

### ApoE genotyping

#### Sanger sequencing

Genomic DNA was isolated using the phenol/chloroform method [24]. The ApoE isoforms: E2 (e2/e3, e2/e2), E3 (e3/e3, e2/e4), E4 (e3e4/, e4/e4) were genotyped in 1002 cases using PCR-Sanger sequencing. The primers for sequencing were described in Table 2. PCR amplification reactions were performed in a final 25 μL containing 50 ng of genomic DNA, 5 pM of each primer, 5 mM dNTPs, 0.5 μL High-GC polymerase (Gen Star Co. Ltd.), 1 μL DMSO, 10 μL buffer and 10.5 μL sterile deionized water. Amplification was performed in a PCR Cycler GT 9612 (BIO-GENER, Hangzhou, China) following the procedure: 95 °C 12 min, 94 °C 1 min, 72 °C 1 min; 95 °C 1 min, 64.8 °C 1 min, 72 °C 1 min in 30 cycles; 72 °C 10 min. PCR product was directly sequenced in the 3730XL sequencer (ABI, Thermo Fisher Scientific, Waltham, MA, US).

**Table 2:** Primers for KASP and Sanger sequencing

Primers	Primer sequence (5′-3′)
1. Sequencing primer-Forward	AACAACTGACCCCGGTGGCG
2. Sequencing primer-Reverse	ATGGCGCTGAGGCCGCGCTCGG
3. RS429358-primer-FAM	CGCGGACATGGAGGACGTGT
4. RS429358-primer-HEX	GCGGACATGGAGGACGTGC
5. RS429358-primer-Common	CTCGCCGCGGTACTGCACC
6. RS7412-primer-FAM	GATGCCGATGACCTGCAGAAGT
7. RS7412-primer-HEX	ATGCCGATGACCTGCAGAAGC
8. RS7412-primer-Common	CCCGGCCTGGTACACTGCC

Primers “1, 2” for Sanger sequencing, “3, 4, 5” for KASP in rs429358 genotyping, and “6, 7, 8” for KASP in rs7412 genotyping

#### KASP method

**Fig. 1:**
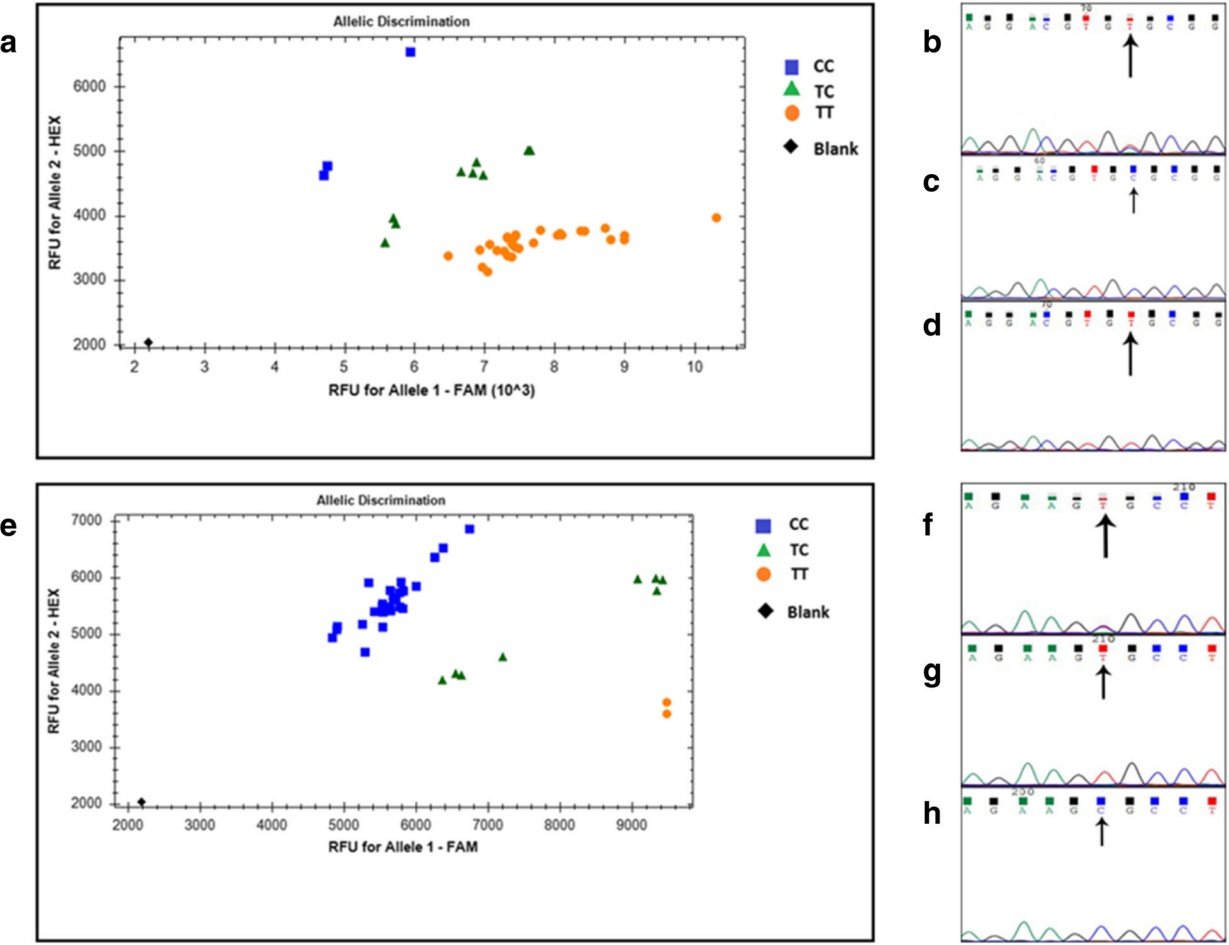
Plots of rs429358 and rs7412 by KASP method and sequencing KASP method for rs429358 (**a**) and rs7412 (**e**), and sequencing results of rs429358 (**b**, **c**, **d**) and rs7412 (**f**, **g**, **h**); **d**: rs429358 T/T (wild type, homozygous); **g**: rs7412 T/T (homozygous); **c**: rs429358 C/C (homozygous); **h**: rs7412 C/C (wild type, homozygous); **b**: rs429358 T/C (heterozygous); **f**: rs7412 T/C (heterozygous).

About 104 cases were randomly chosen for establishing the KASP genotyping method (Fig. 1). The reaction system concluded 50 ng genomic DNA, 5 μL master mix (LGC Genomics, Beverly, MA), 0.14 μL primers (synthesized in LGC Genomics). KASP primers for each SNP were listed (Table 1). KASP reactions were performed in the CFX-connect (Bio-Rad, US) by the standard protocol (LGC Genomics, Beverly, MA) with 96-well plate: 94 °C 15 min; and then 10 cycles of 94 °C 20 s, 61 °C 60 s (drop – 0.6 °C / per cycle); 26 cycles of 94 °C 20 s and 1 min at 55 °C; at last the read step at 37 °C for 1 min. Data were analyzed using the Bio-Rad CFX Manager Software (version 3.1).

#### Statistical analysis

In order to carry out the association analysis between SNPs and statin therapy, “validness” was set as the LDL-C reduction percentage equaled or greater than 25%, otherwise was “voidness” [25].

Continuous variables with skewed data were expressed as median (M, P25~P75) unless otherwise indicated, normal distribution data was assessed using Kolmogorov-Smirnov test, Mann-Whitney U test was used for comparisons of lipid levels before/after statin treatment. The influences of the ApoE alleles (e2/e3/e4) on the response to statins were assessed by analysis of the Kruskal-Wallis test among three ApoE isoforms. The reduction percentages were calculated as:

(*baseline lipids leve* ‐ *after statin therapy lipids level*)/(*baseline lipids level*) ∗ *100*%

All the statistical analysis above was performed using SPSS 22.0 (IBM Corporation, Armonk, New York, US). Multifactor-dimensionality reduction (MDR) method was performed to investigate gene-environmental interactions [26], such as interactions among genes, smoking, alcohol drinking, and T2DM. Best model with maximization of cross-validation consistency (CVC) was selected, and *P* values of prediction accuracy were determined by 100 permutations. The *P* values of less than 0.05 were considered statistically significant.

## Results

### Clinical characteristics of patients

The results of ApoE genotyping using KASP method or sequencing were consistent with each other (Fig. 1), indicating the KASP method was reliable and the ApoE genotyping results were convincing. The distribution of the ApoE genotypes in the total of 1002 individuals was shown (Table 3). The genotype e3/e3 was most frequent, and two genotypes (e2/e3, e3/e4) had a similar frequency.

**Table 3:** Frequencies of ApoE genotypes and alleles in the study population

Genotype	e2/e2	e2/e3	e2/e4	e3/e3	e3/e4	e4/e4	e2	e3	e4
Frequency (n)	2	162	14	633	177	14	180	1605	219
Percent (%)	0.2	16.2	1.4	63.2	17.6	1.4	8.98	80.09	10.93

### Lipid profile related to sex, diabetes and lifestyle

**Fig. 2:**
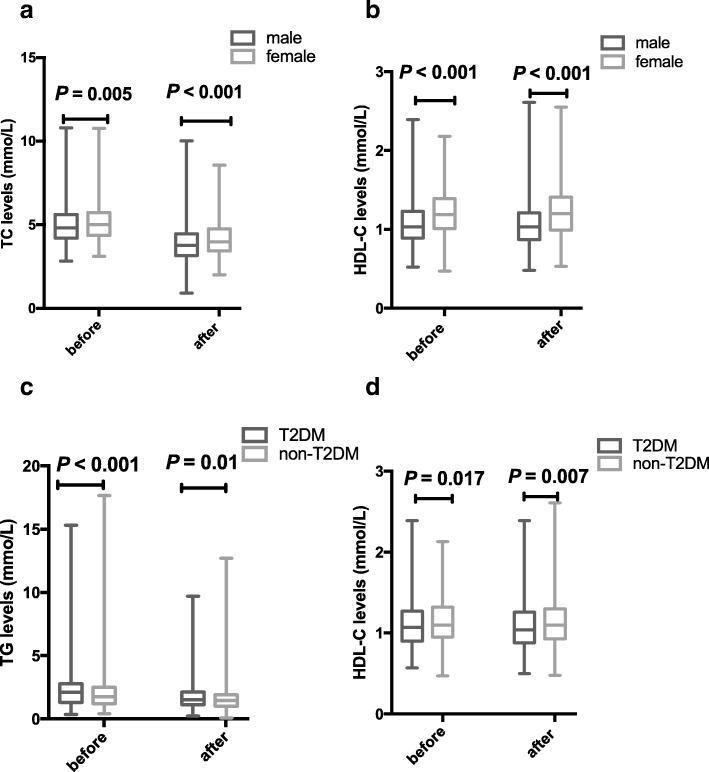
Changes of lipids profiles with statin treatment The difference between male and female of statin therapy in TC (**a**) and LDL-C (**b**) levels. The difference between T2DM and non-T2DM of statin therapy in TG (**c**) and HDL-C (**d**) levels.

Females have significantly higher levels in TC and HDL-C either in the baseline levels (*P* = 0.005, < 0.001, respectively; Fig. 2a) or after statin treatment (*P* < 0.001, both; Fig. 2b) than males. However, the lipid reduction percentage after statins treatment has no significant difference between men and women.

The TG and HDL-C baseline levels of dyslipidemia patients with T2DM were greater than the patients without diabetes (*P* < 0.001, = 0.017, respectively). And the two groups also had a significant difference after statin therapy in TG and HDL-C levels (*P* = 0.01, 0.007, respectively; Fig. 2c, d). There was no significant lipid reduction percentage between the two statin drugs (atorvastatin/rosuvastatin).

**Fig. 3:**
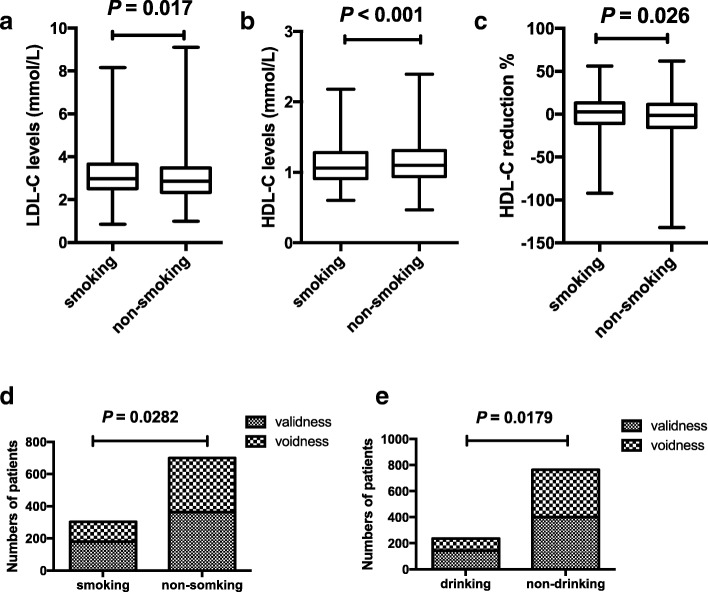
Associations between lifestyles and statins responses The difference between smoking patients and non-smoking patients in LDL-C (**a**) and HDL-C (**b**) baseline levels. The difference in he HDL-C response to statins in smoking and non-smoking groups (**c**). Associations between lifestyles and statins responses to LDL-C. (**d**: smoking status, **e**: alcohol drinking status). “validness” means the LDL-C reduction percentage ≥ 25%, and “voidness” means the LDL-C reduction percentage < 25%.

Patients with smoking had a higher LDL-C baseline and a lower HDL-C baseline and lower elevation of HDL-C level after statins treatment than the non-smoking patients (*P* = 0.017, < 0.001 and = 0.026 respectively; Fig.3a, b, c). And the cases with effective LDL-C lowering response, was statistically more in patients without smoking and alcohol drinking status compared with patients in smoking and alcohol drinking status (Fig. 3d, e).

### Statins response associated with ApoE genotypes

**Fig. 4:**
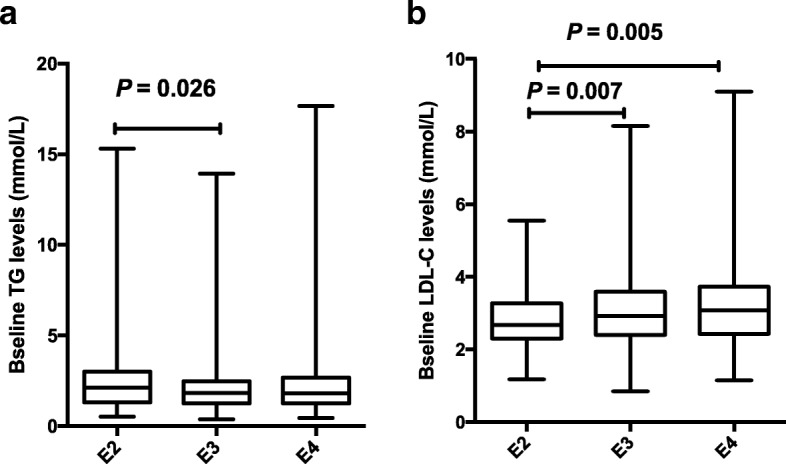
Lipids baseline levels among ApoE genotypes The association of ApoE polymorphisms and baseline levels in TG (**a**) and LDL-C (**b**).

**Fig. 5:**
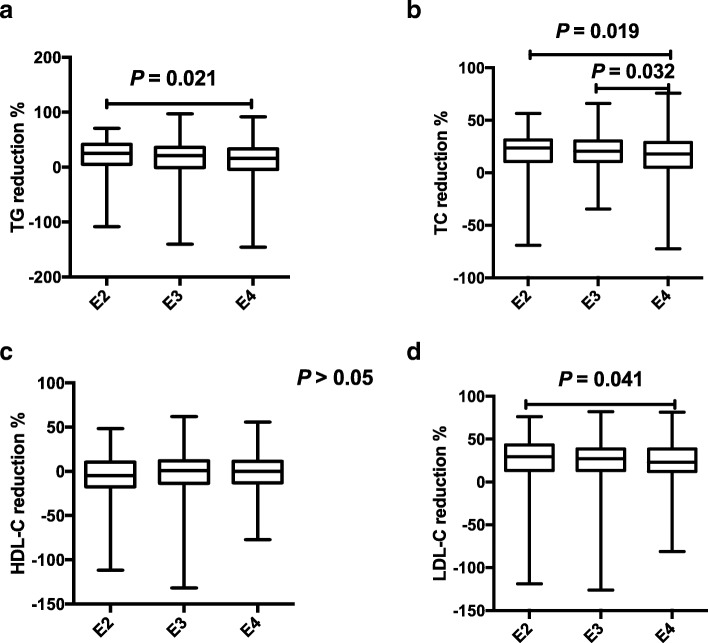
Associations between statin response and ApoE variations The relationships between ApoE polymorphisms and lipids reduction with statin therapy in TG (**a**), TC (**b**), HDL-C (**c**) and LDL-C (**d**).

Distributions of lipids profile in different ApoE isoforms and the response to statins were described (Table 4). A total of 1002 patients were divided into three groups by the ApoE isoforms: E2 (e2/e3, e2/e2), E3 (e3/e3, e2/e4), E4 (e3e4/, e4/e4) phenotype groups. The E2 group had the highest TG baseline, and there was a significant difference between E2 and E3 groups (*P* = 0.026, Fig. 4a). The E4 group had the highest LDL-C baseline, and the significant difference was found among E2, E3, and E4 (*P* = 0.007, 0.005, respectively, Fig. 4b). The reduce percentages of TG, TC, and LDL-C after statins treatment had a statistical difference among E2, E3, E4 groups, showing a significant decrease gradually from E2 to E3 and further to E4 groups (Fig. 5a, b, d). However, no significant difference was found in HDL-C reduction (*P* > 0.05; Fig. 5c).

**Table 4:** Distribution of lipids profile in different ApoE isoforms and the response to statins

Lipid levels	E2 (*n* = 164)	E3 (*n* = 647)	E4 (*n* = 191)	*P* value
before				
TG	2.14 (1.30–3.00)	1.83 (1.24–2.48)	1.81 (1.24–2.68)	0.031
TC	4.80 (4.31–5.44)	4.87 (4.22–5.68)	5.01 (4.27–5.69)	–
HDL-C	1.06 (0.92–1.32)	1.09 (0.94–1.31)	1.11 (0.94–1.27)	–
LDL-C	2.68 (2.29–3.28)	2.92 (2.40–3.59)	3.08 (2.43–3.73)	0.003
after				
TG	1.54 (1.03–2.14)	1.48 (1.08–1.91)	1.47 (1.05–2.13)	–
TC	3.64 (3.12–4.37)	3.84 (3.25–4.51)	4.09 (3.33–4.95)	0.002
HDL-C	1.11 (0.90–1.32)	1.08 (0.91–1.29)	1.08 (0.91–1.27)	–
LDL-C	1.89 (1.50–2.35)	2.08 (1.68–2.68)	2.25 (1.76–2.87)	< 0.001
reduction %				
TG	25.20 (5.30–41.57)	21.03(−0.68–35.84)	16.02(− 4.17–33.33)	0.021
TC	23.56 (10.73–31.24)	20.49 (10.70–30.47)	17.92 (5.20–28.92)	0.012
HDL-C	−4.65(−17.56–10.58)	0.85(− 13.51–11.94)	0(−12.94–11.38)	–
LDL-C	29.41 (13.47–43.14)	27.25 (13.53–38.70)	23.05 (12.47–38.46)	0.042

Values are expressed as M (p25, p75); TC: total cholesterol; LDL-C: low-density lipoprotein cholesterol; HDL-C: high-density lipoprotein cholesterol; TG: triglycerides; *p*-values from K-W test, “-” means *P* > 0.05. E2: e2/e2 and e2/e3, E3: e3/e3 and e2/e4, E4: e3/e4 and e4/e4; “before”: before statin therapy; “after”: after statin therapy

### MDR analysis of gene-lifestyle interaction

**Fig. 6:**
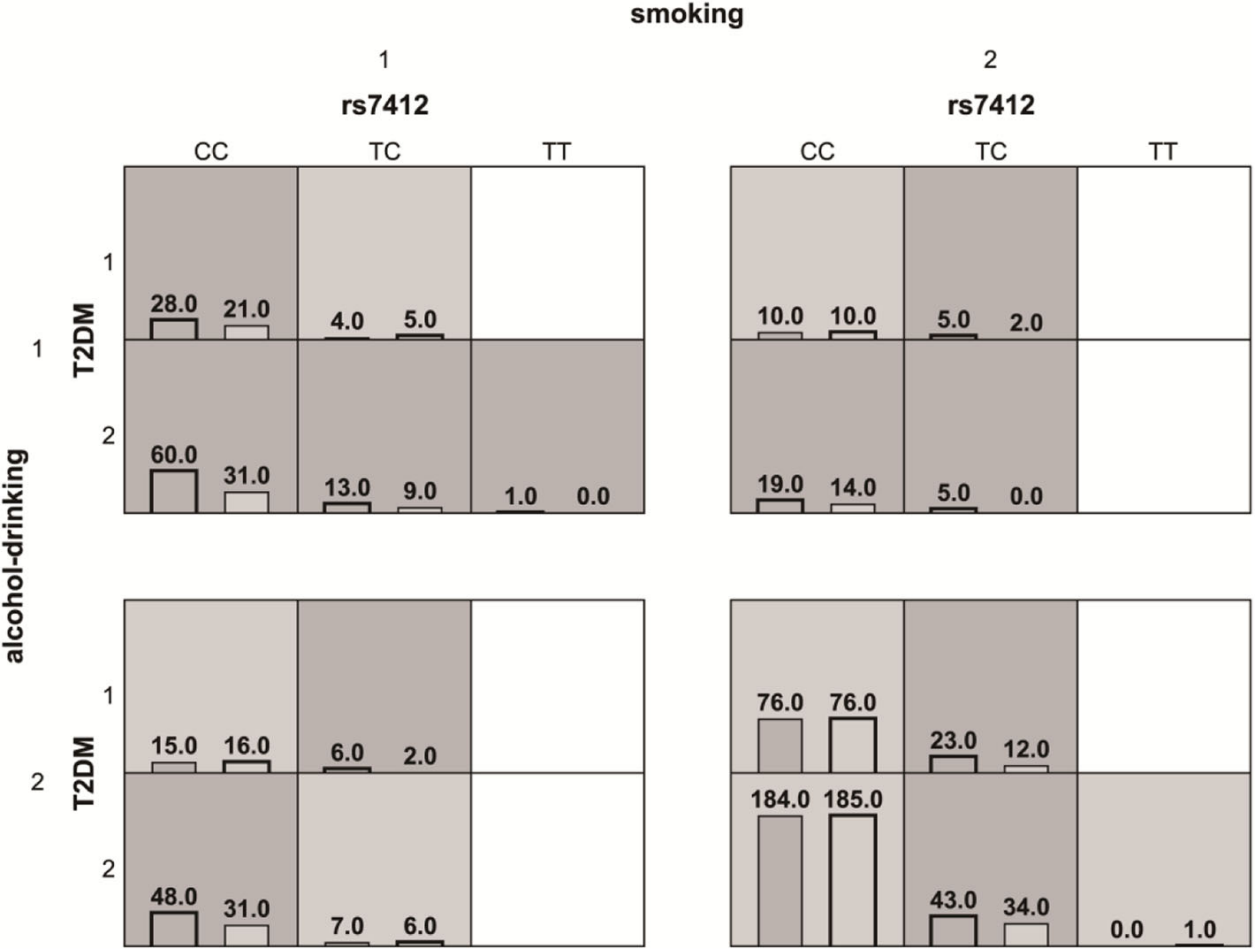
Distribution of high-risk and low-risk genotypes in the best four-locus model Distribution of high-effective and low-effective models associated with statin response to LDL-C among rs7412, smoking status, alcohol drinking, and T2DM. The numbers of “effective” patients (left) and “non-effective” (right) are shown in cells. Dark gray indicates high-effective models, gray cells indicate low-effective models. Blank cells indicate no subjects. “1” means have the habit of smoking/drinking or the underlying disease of T2DM, “2” means negative of them.

One four-locus model rs7412-smoking-alcohol-drinking-T2DM had a maximum testing accuracy of 54.94% and a maximum cross-validation consistency (100/100) that was significant (*P* = 0.047) after permutation testing (Table 5). The four-locus rs7412 combinations risk factors for each multilocus-factor combination were shown in Fig. 6. The combinations of rs7412 T/C-no smoking-no drinking-noT2DM had a high effect in LDL-C lowering; fewer patients with smoking or alcohol drinking achieved a better therapeutic effect when they carried rs7412 T/C or C/C genotypes, such as patients in E4 groups. The models of rs429358-smoking-alcohol drinking-T2DM did not get a significant *P*-value after permutation test, even if the CVC was (100/100).

**Table 5:** Multifactor dimensionality reduction analysis of rs7412 and smoking, alcohol drinking and T2DM

Model	Testing accuracy	CVC	*P*-value
smoking	0.505	90/100	0.9241
rs7412, smoking	0.5492	100/100	0.049
rs7412, smoking, T2DM	0.5174	82/100	0.8132
rs7412, smoking, alcohol drinking, T2DM	0.5494	100/100	0.047

## Discussion

In this study, we investigated the association between two common ApoE polymorphisms (rs7412 and rs429358) and baseline lipid profiles and statins response in dyslipidemia ASCVD patients classified with clinical E2/3/4 phenotypes.

As we are known, ApoE plays an important role in lipids clearance and lipoprotein metabolism [27]. The ApoE gene has three common alleles e2, e3 and e4 encoded three amino acid isoforms: ApoE2, E3 and E4, therefore, producing six common genotypes (e2/e2, e2/e3, e3/e4, e4/4, e3/e3, e2/e4.) [28]. ApoE4 isoform has a higher affinity for receptors of low-density lipoprotein receptor (LDLR) family, such as LDLR, very low-density lipoprotein (VLDL) and LDLR-related protein (LRP) and apoE receptor 2, which result in higher uptake or degradation of apoE. This would generate lower cholesterol efflux activity, which would produce elevated cholesterol levels in circulation and lead to a higher risk of cardiovascular diseases [27]. E2 amino acid isoform has a low affinity with VLDL, which induced an increased VLDL in plasma, that is why lots of patients with type III hyperlipidemia (HLP III) were e2 homozygous [29]. However, people with e2 homozygotes got type III HLP less than 10%, indicated that other genetic factors might be contributing to this disease in its progression [30].

We got some coincident results with the most previous study. First, TG baseline was higher in dyslipidemia patients with T2DM compared to those without T2DM [31]. Second, female patients had higher baselines of TC, LDL-C, and HDL-C. Third, there were significantly increased baselines of LDL-C from E2 to E3 and further to E4 groups.

Significant differences were also found in lipids response to statins among the three ApoE phenotypes in this study, E2 phenotype groups had a better lowering cholesterol effect than E4 phenotype groups. Although the HDL-C increment percentage among the three groups had no statistically significant difference, e2 carriers showed a better elevation. As a meta-analysis of statins response to ApoE polymorphisms that e4 carriers had a worse response to statin treatment than that of e2 carriers, mainly in Caucasian [32]. Actually, Statins response to lipids associated with ApoE polymorphisms has been reported in several studies, but the results were inconsistent [17, 18, 32]. Ethnicities, therapy plan, statistical method selection and other genes associated with cholesterol biosynthesis and metabolism, might be possible causes of variation in response to statins. Since the dosage of statins in China is much lower compared that in the western country and Chinese might have a different response to statin therapy, the association study between ApoE polymorphisms and statin effects in Chinese is required.

Consistent with other investigations, the patients with smoking had a significantly higher baseline LDL-C levels and lower HDL-C levels than the non-smoking people and the non-smoking people also had a higher HDL-C level than the smokers after statins therapy. These discoveries may be revealing that smoking is a negative factor in lipid-lowing therapy of statins, which indicate lifestyles management combined with statin therapy is necessary. According to our study, obviously different statins response to LDL-C in smoking and alcohol drinking habits was shown by different statistical methods. More patients with the SNP site rs7412 TC/CC in good living habits could achieve the therapy goal. And these results also indicate that medium dosage of statins to individuals with unhealthy lifestyles or underlying diseases might be ineffective in E4 patients.

In addition, today real-time quantitative PCR (q-PCR) and hybridization techniques were applied for ApoE genotyping in most clinic labs. However, KASP method established in our study has been verified using Sanger sequencing and was more economic compared to q-PCR methods while more convenient compared to hybridization methods [19, 33]. The KASP tests could be finished in 1 PCR tube for 1 SNP after reaction results could be read directly with no more steps. Owing to less labeled-specific allele primers, the total price of the detections was cost-effective in large-scale screening which was usual in research investigation. And this method has developed into a global benchmark technology in SNP genotyping platform [34], it would be acceptable for clinical diagnostic use in further medical research.

In this study, 1002 Chinese ASVCD patients with statin treatment in dysemia were recruited, it was a larger population study compared to other ethnicity studies previously [17, 18]. Our research could be providing a reference for the clinical applications of statins to kinds of patients in drug selecting and individual-based treatment.

Some limitations of our study merit consideration. Firstly, due to the lack of the RNA samples, analysis the relationships between ApoE polymorphisms and its protein expression before and after statins therapy was not available. Secondly, exploring the mechanisms of the SNPs in the statins therapy would be necessary for further study. At last, the lipids regulation of statins was a complex process involved pharmacodynamics and pharmacokinetics with kinds of organs and genes, comprehensive furthermore exploration on gene panels is needed.

## Conclusions

This study confirms the strong relationships between ApoE polymorphisms and lipids baseline levels, so as to the lipids responses to statins among ApoE protein isoforms in Chinese dyslipidemia ASCVD patients, and the self-build KASP ApoE genotyping method was reliable with great application prospect. Enhance drugs dose and better living habits management in ApoE4 phenotype patients could be more efficient in the clinical treatment in China.

## Abbreviations

ApoE: Apolipoprotein E
ASCVD: Arteriosclerotic cardiovascular diseas
CVC: cross-validation consistency
HDL-C: High-density lipoprotein cholesterol
HLP III: Type III hyperlipidemia
HRM: high resolution melting
KASP: Kompetitive Allele Specific PCR
LDL-C: Low-density lipoprotein cholesterol
MDR: Multifactor-dimensionality reduction
PCR: Polymerase chain reaction
Q-PCR: Real-time Quantitative PCR
SNP: single nucleotide polymorphism
T2DM: Type 2 diabetes mellitus
TC: Total cholesterol
TG: Triglyceride
VLDL: very low-density lipoprotein
